# Prion Infection Impairs Cholesterol Metabolism in Neuronal Cells*[Fn FN2]

**DOI:** 10.1074/jbc.M113.535807

**Published:** 2013-11-26

**Authors:** Huanhuan L. Cui, Belinda Guo, Benjamin Scicluna, Bradley M. Coleman, Victoria A. Lawson, Laura Ellett, Peter J. Meikle, Michael Bukrinsky, Nigora Mukhamedova, Dmitri Sviridov, Andrew F. Hill

**Affiliations:** From the ‡ Baker Heart and Diabetes Institute, Melbourne, Victoria 8008, Australia,; the § Department of Biochemistry and Molecular Biology, Bio21 Molecular Science and Biotechnology Institute, and; ¶ Department of Pathology, University of Melbourne, Melbourne, Victoria 3010, Australia, and; the ‖ Department of Microbiology, Immunology, and Tropical Medicine, George Washington University, Washington, D. C. 20854

**Keywords:** ABC Transporter, Cholesterol, Lipid Raft, Neurodegenerative Diseases, Prions

## Abstract

Conversion of prion protein (PrP^C^) into a pathological isoform (PrP^Sc^) during prion infection occurs in lipid rafts and is dependent on cholesterol. Here, we show that prion infection increases the abundance of cholesterol transporter, ATP-binding cassette transporter type A1 (ATP-binding cassette transporter type A1), but reduces cholesterol efflux from neuronal cells leading to the accumulation of cellular cholesterol. Increased abundance of ABCA1 in prion disease was confirmed in prion-infected mice. Mechanistically, conversion of PrP^C^ to the pathological isoform led to PrP^Sc^ accumulation in rafts, displacement of ABCA1 from rafts and the cell surface, and enhanced internalization of ABCA1. These effects were abolished with reversal of prion infection or by loading cells with cholesterol. Stimulation of ABCA1 expression with liver X receptor agonist or overexpression of heterologous ABCA1 reduced the conversion of prion protein into the pathological form upon infection. These findings demonstrate a reciprocal connection between prion infection and cellular cholesterol metabolism, which plays an important role in the pathogenesis of prion infection in neuronal cells.

## Teaser

**Background:** Cholesterol plays an important role in the pathogenesis of neurodegenerative diseases.

**Results:** Prion infection increased abundance of cholesterol transporter ABCA1 but reduced its functionality. Stimulation of ABCA1 reduced the conversion of prion into the pathological form.

**Conclusion:** ABCA1 plays a key role in pathogenesis of prion disease.

**Significance:** There is a reciprocal connection between prion infection and cholesterol metabolism.

## Introduction

Cholesterol has increasingly been recognized to play an important role in the pathogenesis of many neurodegenerative diseases. Dysregulation of cholesterol metabolism in the brain has been implicated in the pathogenesis of Niemann Pick type C disease (1), Alzheimer disease, and prion disease (2). Prion diseases, or transmissible spongiform encephalopathies, are fatal neurodegenerative disorders affecting humans and animals. A key event in the pathogenesis of prion disease is the conversion of the host-encoded cellular prion protein (PrP^C^)^7^ to a pathological misfolded isoform (PrP^Sc^). PrP^C^ is enriched with an α-helical structure, is monomeric, and is susceptible to protease digestion, whereas PrP^Sc^ mainly consists of a β-sheet structure that can form insoluble protease-resistant deposits in the brain (3). The exact cellular conversion site and molecular mechanism(s) involved in the conversion of PrP^C^ to PrP^Sc^ are still unknown; however, it has been suggested that conversion takes place in lipid rafts (4, 5). There are several lines of evidence that support this as follows: (i) depletion of cellular cholesterol from the plasma membrane diminishes PrP^Sc^ formation (5, 6); (ii) PrP^Sc^ and PrP^C^ are both glycosylphosphatidylinositol-anchored proteins residing in the lipid rafts (5, 7); (iii) *in vitro*, membrane-associated PrP^C^ is resistant to conversion unless phosphatidylinositol-specific phospholipase C is added to the reaction promoting insertion of the infectious PrP^Sc^ into the rafts to initiate conversion (4); (iv) the modification of properties of lipid raft domains significantly inhibits PrP^Sc^ propagation (8).

Lipid rafts are highly dynamic, short lived microdomains found in the plasma membrane and enriched with cholesterol and sphingolipids. Lipid rafts have also been implicated as a platform for a number of key cellular pathways, such as protein sorting, membrane trafficking, signal transduction, and activation of the immune response (9, 10). The abundance and integrity of these microdomains are highly dependent on the availability of cholesterol (10).

Cholesterol is required for PrP^C^ cell surface expression and stabilization (11), and cholesterol depletion inhibits the replication of the pathological isoform in prion-infected cells by disrupting the trafficking of PrP^C^ to lipid rafts (6). Cholesterol biosynthesis has been shown to be enhanced in prion infection, which was associated with increased levels of cellular cholesterol (12), suggesting a link between cholesterol metabolism and prion propagation. Treatment of prion-infected mice with inhibitors of cholesterol biosynthesis prolonged the latent stage of infection, reduced neuronal loss, and significantly delayed death (13). Collectively, these findings demonstrate that not only prion replication is dependent on the availability of cholesterol, but prions may be capable of manipulating cholesterol metabolism to meet their requirement for cholesterol. Paradoxically, it was reported that prion infection also increases the abundance of ATP-binding cassette transporter A1 (ABCA1) (14). Outside the brain, the main function of ABCA1 is to facilitate cholesterol removal from cells to the lipid-poor acceptor, apolipoprotein A-I (apoA-I). Increased abundance of ABCA1 is generally associated with enhanced reverse cholesterol transport and a reduction of cellular cholesterol content (15). Cholesterol homeostasis in the CNS is separated from the rest of the body as the blood brain barrier does not permit lipoprotein permeability. Within the brain, ABCA1, along with another transporter, ATP-binding cassette transporter G1 (ABCG1), transfers cholesterol and phospholipids to the lipid acceptor, apolipoprotein E (apoE)-containing lipoproteins. ApoE is produced by astrocytes (16, 17) and contributes to regulation of cholesterol homeostasis in neuronal cells.

Given the importance of cholesterol in the pathogenesis of prion disease and the key role of ABCA1 in maintaining cholesterol homeostasis, we investigated the impact of prion infection on the regulation, trafficking, and function of ABCA1. We found that prion infection post-transcriptionally inhibits the ABCA1-dependent cholesterol efflux pathway by redistributing ABCA1 away from the plasma membrane and lipid raft microdomains, ultimately leading to increased amounts of free cholesterol within prion-infected cells. Conversely, overexpression of ABCA1 in prion-infected cells significantly reduced the abundance of PrP^Sc^, demonstrating a key involvement of this pathway in prion propagation. These findings reveal a novel element of pathogenesis of prion disease that could potentially be targeted by therapeutic approaches.

## EXPERIMENTAL PROCEDURES

### 

#### 

##### Cells

Three murine cell models were used as follows: the murine hypothalamic cell line GT1-7 (18), the murine neuroblastoma cell line N2a, and murine fibroblasts 3T3. Neuronal cells were infected with the mouse adapted human prion strain M1000, and the efficiency of infection was monitored every five passages; both cell lines were strongly infected during the course of experimentation (data not shown). The M1000 prion strain was prepared from brains of terminally sick BALB/c mice infected with the mouse-adapted human Fukuoka-1 prion strain (19). All experiments were performed within the first 15 passages following infection with prions. Cells were infected as described previously (20). For sodium pentosan polysulfate (PPS) treatment, mock- and prion-infected GT1-7 cells were treated with PPS (50 μg/ml) or vehicle continuously for 8 days. The cells were passaged every 3 days, and treatment was repeated. 3T3 mouse fibroblast cells were infected using rabbit kidney 13 (RK13) epithelial cell lysates, which have been infected with M1000 prion strain, as described previously (21). 3T3 cells were transfected with mouse ABCA1-specific siRNA (siRNA^ABCA1^) or scrambled siRNA (siRNA^C^) (Ambion). Transfection was performed using Lipofectamine RNAiMAX (Invitrogen) according to the manufacturer's protocol. Overexpression of human ABCA1 in 3T3 cells was achieved by transfecting the cells with human ABCA1-GFP plasmid, using Lipofectamine LTX plus reagent (Invitrogen) according to the manufacturer's protocol.

##### Cell Blot Assay

Cells were plated and grown to confluence on plastic coverslips (Nunc) and transferred on to a nitrocellulose membrane, followed by proteinase K digestion and immunostaining using anti PrP antibody (ICSM-18) as described previously (22).

##### Animals

For the time course analysis, BALB/c mice (6–8 weeks of age) were inoculated in the left parietal region under methoxyflurane inhalational anesthesia with a 1% (w/v) homogenate of M1000 infected brain tissue as described previously (23). Mice were anesthetized (methoxyflurane) and euthanized at predetermined time points (3, 6, 10, 13, and 17 weeks post-inoculation) or when they exhibited clear signs of terminal prion disease. Histology (hematoxylin/eosin staining) and immunohistochemistry using polyclonal anti-ABCA1 antibody (Abcam) were performed on formalin-fixed tissues. Homogenates were prepared in PBS containing complete protease inhibitor mix and normalized for equivalent protein. Brain lysates were prepared as described previously (20). Immunohistochemistry was performed at the Animal Facility and Histology Core Services at the Melbourne Brain Centre (Melbourne, Victoria, Australia).

##### Acceptors

High density lipoprotein (HDL) (1.083 < *d* <1.21 g/liter) was isolated from frozen human plasma by sequential centrifugation in KBr solutions. ApoE discs (reconstituted HDL based on apoE) were prepared with recombinant apoE protein, cholesterol, and 1-palmitoyl-2-oleylphosphatidylcholine (POPC), using the cholate dialysis method as described (24). The POPC/cholesterol/apolipoprotein molar ratio of the apoE discs was 114:13:1. Concentration of the acceptors was expressed as protein concentration. Methyl β-cyclodextrin·cholesterol complexes were prepared as described previously (25) and used at the final concentration 5 mm.

##### Cholesterol Efflux Assay

Cholesterol efflux was performed as described previously (26). Concentrations of the acceptors were as follows: apoA-I, 30 μg/ml; HDL, 40 μg/ml, and apoE discs, 15 μg/ml. Duration of the efflux incubation was 4 h, and LXR agonist TO-901317 was used at final concentration of 4 μm.

##### Real Time Quantitative PCR

Cells were seeded in a 6-well tissue culture plates and treated or untreated with 4 μm TO-901317 for 18 h. Total RNA was isolated using the TRIzol method. cDNA were synthesized from 2 μg of RNA with random primers using High Capacity cDNA reverse transcription kit (Invitrogen) according to the manufacturer's recommendation. Specific primers for each gene (*Abca1* (Mm00442626_m1), *Abcg1* (Mm00437390_m1), *Ldl-r* (Mm00440169_m1), and *Prnp* (Mm00448389_m1)) were from Invitrogen. The PCRs were done in triplicate and normalized to *gapdh* mRNA. The relative amount of mRNA was calculated by using the comparative threshold cycle (ΔΔ*C_T_*) method.

##### Confocal Microscopy

Cells were seeded in 8-well chamber slides (Thermo Scientific or Ibidi) and maintained at 37 °C for 24 h. The cells were washed with PBS, fixed with 4% paraformaldehyde for 15 min at room temperature, washed with PBS, and permeabilized with 0.1% Triton X-100 for 10 min. After blocking with 10% goat serum in PBS for 1 h, primary antibodies (monoclonal anti-ABCA1 antibody and polyclonal anti-LAMP1 antibody) in PBS containing 1% goat serum were added to the cells for 18 h at 4 °C. Cells were then washed with PBS, incubated with appropriate fluorescent-conjugated secondary antibodies (Invitrogen), and counter-stained with 4′,6′-diamidino-2-phenylindole (DAPI; Sigma; 1 μg/ml) for 2 h at room temperature. Confocal microscopy performed using a Leica TCS SP2 imaging system.

##### Cell Surface ABCA1 Assay

Cells were grown in a 75-cm^2^ flask and activated with LXR agonist TO-901317 (4 μm) for 18 h. Cells were washed with cold PBS and incubated for 30 min at 4 °C in PBS with Sulfo-NHS-SS-Biotin (Pierce) (final concentration 0.5 mg/ml). Cells were then washed twice with cold quench buffer (50 mm Tris, 0.1 mm EDTA, 150 mm NaCl) and once with cold PBS. Cells were scraped into PBS containing complete protease inhibitor mixture (Roche Applied Science) and lysed with 5 mm Tris, pH 7.5. Large debris removed by low speed centrifugation and cellular membrane fraction was isolated by centrifugation at 100,000 × *g* for 1 h at 4 °C. Pellet was resuspended in a 50 mm Tris, 22 mm mercaptoethanol, 1% Triton X-100 buffer containing complete protease inhibitor mixture. Membrane lysates were mixed with UltraLink Plus immobilized streptavidin beads (Pierce) and incubated for 2 h at 4 °C. After extensive washing with PBS, the beads were incubated with SDS-PAGE sample buffer containing 50 mm DTT and heated at 50 °C for 30 min. Beads were then pelleted by centrifugation. Samples of supernatant were analyzed using Western blot. To trace ABCA1 internalization, biotinylated cells were returned to 37 °C and incubated for 30 min. Biotin from biotinylated proteins remaining at the cell surface was cleaved off by incubating cells with 50 mm tris(2-carboxyethyl)phosphine (Sigma) in Tris-based buffer for 30 min at 4 °C. The remaining biotinylated ABCA1 was considered the internalized portion. The cells were lysed with RIPA buffer (Pierce), and protein was mixed with UltraLink Plus immobilized streptavidin beads (Pierce), incubated for 2 h at 4 °C, and processed as described for cell surface ABCA1 assay.

##### Lipid Raft Isolation

Lipid rafts were isolated using a detergent-free method (27). Briefly, cells were grown in a 75-cm^2^ flask and activated with LXR agonist TO-901317 (4 μm) for 18 h prior to collection. Cells were washed with PBS and resuspended in a 20 mm Tris-HCl, pH 7.8, 250 mm sucrose, 1 mm CaCl_2_, and 1 mm MgCl_2_ buffer containing protease inhibitor mixture. Cells were lysed by passing through a 27-gauge needle 20 times. Lysates were pelleted by centrifugation, and supernatant was collected. The remaining cell pellet was lysed by passing through the 27-gauge needle 20 times on ice, and large debris pelleted by centrifugation and supernatant was collected and combined with the first collection. The collected supernatant was combined with 50% OptiPrep density gradient medium to produce a final concentration of 25% and loaded at the bottom of the 8.9-ml ultracentrifuge tube. A 20 to 5% continuous gradient was laid on top of the lysates. Samples were centrifuged for 18 h, 52 × 10^3^ × *g* at 4 °C. After centrifugation, 0.6-ml fractions were collected, and proteins were precipitated using the methanol/chloroform method. Fractions were analyzed by Western blotting.

##### Lipidomics Analysis

GT1-7 cells were collected, resuspended in 0.5 m NaCl, 20 mm Tris, pH 7.0, and cell pellets were sonicated. Lipids were extracted using chloroform/methanol (2:1) from cell lysates (20 μg of cellular protein). Lipid analysis was performed by liquid chromatography, electrospray ionization-tandem mass spectrometry (LC ESI-MS/MS) using a Agilent 1200 liquid chromatography system, and Applied Biosystems API 4000 Q/TRAP mass spectrometer with a turbo-ion spray source (350 °C) and Analyst 1.5 and MultiQuant data systems using a Zorbax C18, 1.8 μm, 50 × 2.1-mm column (Agilent Technologies). Lipid concentrations were calculated by relating the peak area of each species to the peak area of the corresponding internal standard.

##### Other Methods

For PrP^Sc^ detection, cells were collected and lysed, and 100 μg of protein was digested with proteinase K at a final concentration of 25 μg/ml for 60 min at 37 °C. Digestion was stopped by adding 5 mm phenylmethylsulfonyl fluoride to the lysates for 5 min on ice. Brain homogenates were prepared as described previously (19, 20); for Western blots, brain homogenates were diluted in lysis buffer. The following antibodies were used for Western blot analysis: monoclonal anti-ABCA1 antibody (Abcam); polyclonal anti-ABCG1 antibody (Abcam); polyclonal anti-LDL receptor antibody (Abcam); in-house monoclonal anti-PrP antibody L3; and horseradish peroxidase-conjugated anti-IgG (GE Healthcare).

##### Statistics

The data are presented mean ± S.D. of quadruplicate determinations. Statistical significance of the differences was assessed by Student's *t* test.

##### Ethics Statement

Animal experimentation was approved by the Animal Ethics Committee of Melbourne University and conformed to the Guide for the Care and Use of Laboratory Animals (National Institutes of Health).

## RESULTS

### 

#### 

##### Prion Infection Increases Abundance of ABCA1 but Reduces Cholesterol Efflux

**FIGURE 1.**
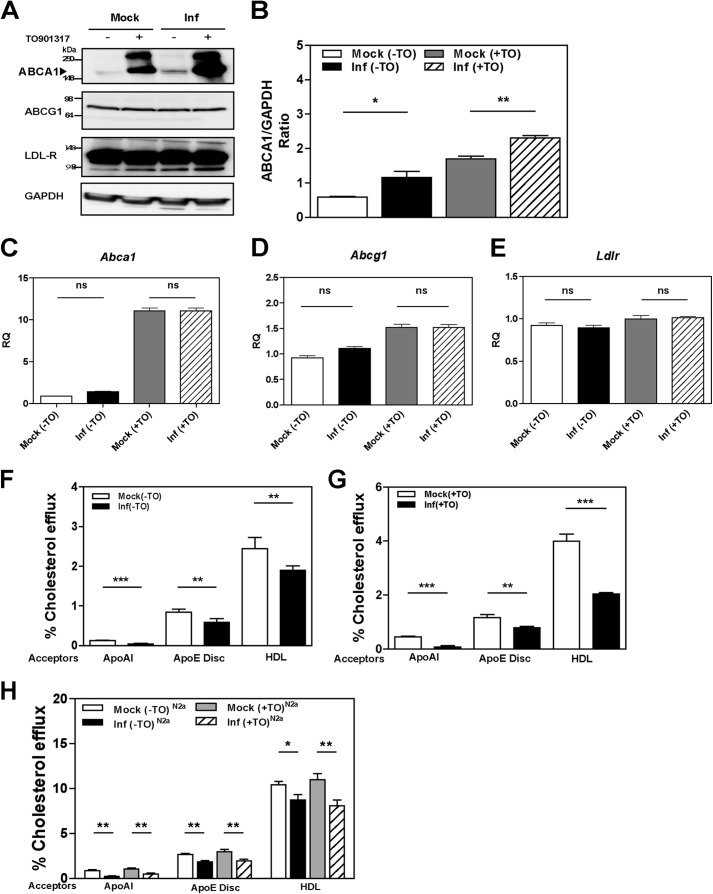
**Prion infection increases ABCA1 protein abundance via a post-transcriptional mechanism but impairs cholesterol efflux.** *A,* mouse GT1-7 cells infected (*Inf*) with prion or mock-infected (*Mock*) were treated or not with LXR agonist 4 μm TO901317 for 24 h. ABCA1, ABCG1, LDL receptor, and GAPDH proteins were analyzed with Western blotting. *B,* densitometric quantification of ABCA1 protein abundance relative to GAPDH in mock- and prion-infected GT1-7 cells. *, *p* < 0.05; **, *p* < 0.01. *C–E,* effects of prion infection on mRNA expression levels of *Abca1* (*C*), *Abcg1* (*D*), and *Ldlr* (*E*) were assessed by RT-PCR. *F,* cholesterol efflux to apoA-I (30 μg/ml), apoE discs (15 μg/ml), and HDL (40 μg/ml) from mock- or prion-infected GT1-7 cells, without LXR agonist treatment. *G,* same as *F*, but cells were treated with LXR agonist. *H,* cholesterol efflux to lipid acceptors apoA-I (30 μg/ml), apoE discs (15 μg/ml), and HDL (40 μg/ml) were measured in mock- and prion-infected N2a cells. Cells were treated (+) or untreated (−) with TO901317 (*TO*). *, *p* < 0.05; **, *p* < 0.01, and ***, *p* < 0.001; *ns,* nonsignificant.

To investigate the effect of prion infection on cellular cholesterol metabolism, we assessed changes in the abundance of the LDL receptor and the two key cholesterol transporters ABCA1 and ABCG1. The third key cholesterol transporter, SR-BI, is not expressed in neuronal cells (28). Prion infection significantly increased the abundance of ABCA1 protein in GT1-7 cells (Fig. 1, *A* and *B*). This was evident in both unstimulated cells and in cells where expression of ABCA1 was stimulated with LXR agonist TO-901317. In contrast, prion infection and LXR agonist had no effects on the protein levels of ABCG1 and the LDL receptor (Fig. 1*A*).

To investigate the mechanism of stimulation of ABCA1 abundance, the expression levels of *Abca1, Abcg1,* and *Ldlr* genes were measured by quantitative real time PCR and normalized to the expression of *Gapdh*. Abundance of *Abca1* mRNA increased by 10-fold following treatment with LXR agonist in both mock- and prion-infected cells (Fig. 1*C*). Prion infection had no significant effect on *Abca1* expression in both LXR agonist-treated and untreated cells. Treatment with LXR agonist caused a modest (50%) increase in the level of *Abcg1* mRNA and had no effect on the level of *Ldlr* mRNA (Fig. 1, *D* and *E*). Prion infection did not influence the expression of *Abcg1* and *Ldlr* with or without treatment with LXR agonist. Collectively, these findings suggest that prion infection specifically enhances the abundance of the ABCA1 transporter via a post-transcriptional mechanism.

The functionality of ABCA1 is reflected in the rate of cholesterol efflux to lipid-free acceptors, mainly apolipoprotein A-I (apoA-I). However, the levels of apoA-I in the CNS are reported to be very low, and the most abundant apolipoprotein found in the CNS is apoE (29). Therefore, we tested the effects of prion infection on ABCA1 functionality by measuring cholesterol efflux to recombinant discoidal lipoproteins composed of apoE and POPC, apoA-I, or HDL. As expected, when cholesterol efflux from nonactivated mock-infected GT1-7 cells was measured, more cholesterol was released to the lipidated acceptors, apoE/POPC and HDL, than to the lipid-free apoA-I (Fig. 1*F*). Unexpectedly, significantly less cholesterol was released from prion-infected cells compared with mock-infected cells to all three acceptors (Fig. 1*F*). When cells were activated with LXR agonist, the percentage of efflux to lipid-free apoA-I, apoE/POPC, and HDL was increased by 200, 30, and 64%, respectively (Table 1). Prion infection resulted in a significant reduction of cholesterol efflux from cells activated with LXR agonist to all three acceptors (Fig. 1*G*). When ABC-dependent efflux (defined as a difference between the efflux from activated *versus* nonactivated cells) was calculated, ABC transporter-specific cholesterol efflux to apoA-I and HDL was reduced by 80% and to apoE/POPC by 45% in prion-infected cells compared with mock-infected cells. Nonspecific efflux (*i.e.* the efflux in the absence of acceptors) was not affected by prion infection (Table 2).

**TABLE 1:** **The effect of the LXR agonist and prion infection on cholesterol efflux from GT1-7 cells**

LXR agonist	Acceptor	Efflux from mock-infected cells	Efflux from prion-infected cells	*p*
		%	%	
(−) TO901317	ApoA-I	0.13 ± 0.01	0.04 ± 0.02	<0.001
	ApoE/POPC	0.89 ± 0.02	0.59 ± 0.09	<0.02
	HDL	2.44 ± 0.28	1.90 ± 0.11	<0.01
(+) TO901317	ApoA-I	0.45 ± 0.03	0.08 ± 0.05	<0.0001
	ApoE/POPC	1.17 ± 0.11	0.80 ± 0.05	<0.001
	HDL	3.99 ± 0.27	2.04 ± 0.05	<0.0001

**TABLE 2:** **Nonspecific cholesterol efflux from GT1-7 cells**

Cells	Cholesterol efflux	
	−TO901317	+ TO901317
	%	
Mock GT1-7	0.536 ± 0.181	0.516 ± 0.235
Infected GT1-7	0.460 ± 0.107	0.460 ± 0.188
*p*	>0.5	>0.5

The findings with GT1-7 cells were confirmed using a second model, mouse neuroblastoma N2a cells. In contrast to GT1-7 cells, LXR agonist did not affect cholesterol efflux from N2a cells to any of the acceptors (Fig. 1*H*). Consistent with the findings on GT1-7 cells, prion infection resulted in a significant reduction in cholesterol efflux to apoA-I, apoE/POPC, and HDL (Fig. 1*H*). Thus, cholesterol efflux from prion-infected cells was inhibited despite the elevated abundance of ABCA1 and unchanged abundance of ABCG1.

##### Prion Infection Increases Abundance of ABCA1 in Vivo

**FIGURE 2.**
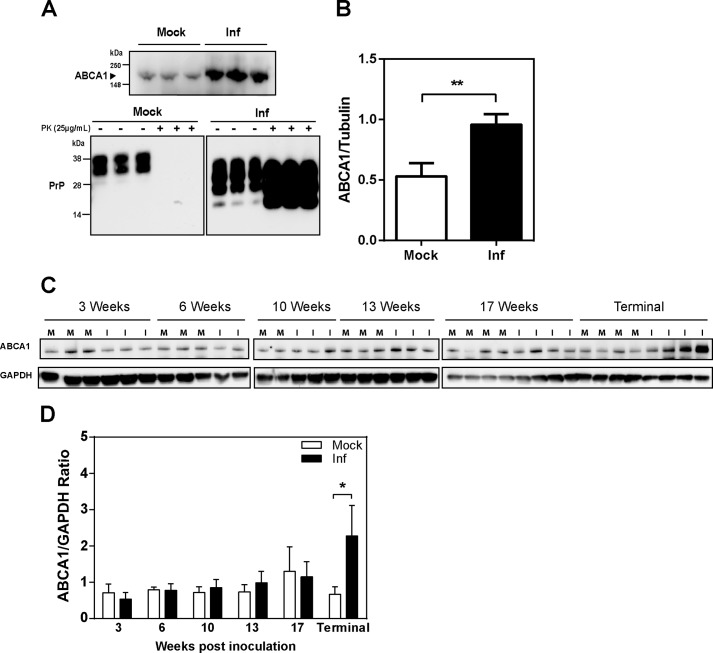
**Prion infection (*Inf*) increases ABCA1 protein abundance *in vivo*.** *A, top panel,* ABCA1 abundance in brains of mock and M1000-infected BALB/c mice. *Bottom panel,* detection of PrP^C^ and PrP^Sc^ from brain homogenates treated (+) or not treated (−) with PK. *B,* densitometric quantitation of Western blots presented in *A*; **, *p* < 0.001. *Inf*, infected. *C,* Western blot analysis of ABCA1 abundance in the brain of prion-infected mice at different stages of infection (weeks after inoculation). *D,* densitometric quantitation of Western blots presented in *C*; *, *p* < 0.001.

To investigate if prion infection affects abundance of the ABCA1 transporter *in vivo*, we compared ABCA1 abundance in brain tissue homogenates from clinically affected prion-infected mice and age-matched noninfected controls. As shown in Fig. 2*A*, with quantitation shown in Fig. 2*B*, ABCA1 abundance was significantly elevated in prion-infected brain samples; the infection in these brain samples was confirmed using Western blotting as shown in the *lower panel* of Fig. 2*A*.

Fig. 2*C* shows abundance of ABCA1 in brain homogenates of mice infected with prions for various periods of time; quantitation of the abundance is shown in Fig. 2*D*. Increased abundance of ABCA1 was only evident at the terminal stage of the infection (Fig. 2, *C* and *D*).

**FIGURE 3.**
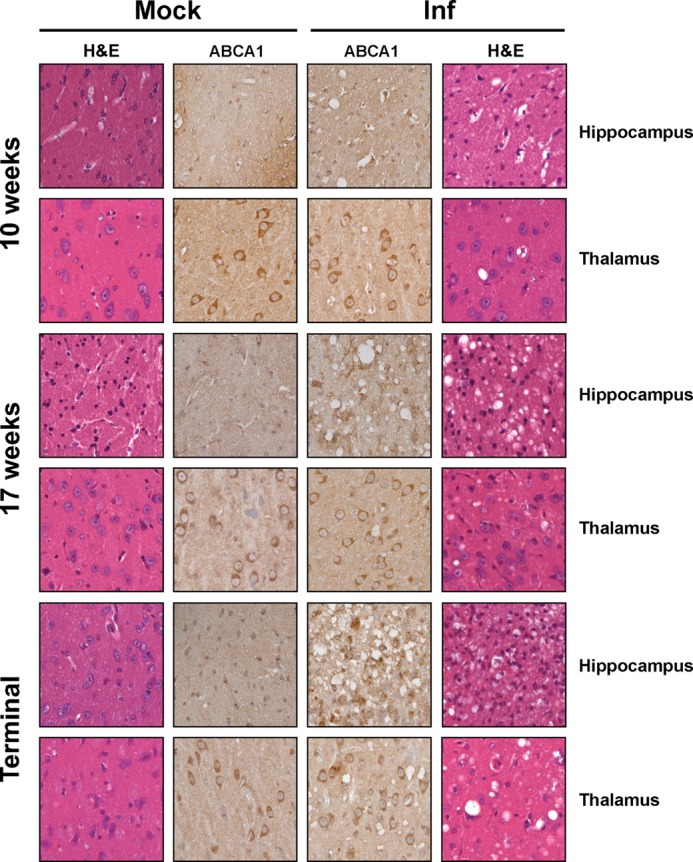
**Changes in ABCA1 expression correlates with degree of pathology associated with prion infection *in vivo*.** Histology (*H&E*) and immunohistochemistry (ABCA1) were performed on sagittal brain sections of mock- and prion-infected mice prepared at the indicated time points (weeks post-inoculation) or when they exhibited signs of clinical prion disease (*terminal*). Images of thalamus and the CA3/fimbria of the hippocampus are shown (*n* ≥2 mice per time point).

Fig. 3 shows histology and immunochemical staining of ABCA1 in sections of the thalamus and hippocampus of mock- and prion-infected mice. It reveals general ABCA1 immunoreactivity in the cell bodies and neuropil of both mock- and prion-infected mice, with a more prominent expression observed in the bodies of cells located in the thalamus. There was no significant change in ABCA1 abundance at 10 weeks post-inoculation, when vacuolation was first observed in the hippocampus and thalamus of prion-infected mice (30). However, a change in ABCA1 localization was observed in the CA3 and fimbria regions of the hippocampus 17 weeks post-inoculation, when the region exhibited clear vacuolation and spongiform change. A change in ABCA1 localization was also observed in cells of the thalamus; this, however, varied with the degree of vacuolation observed.

##### Prion Infection Impairs Cholesterol Efflux by Affecting ABCA1 Localization

**FIGURE 4.**
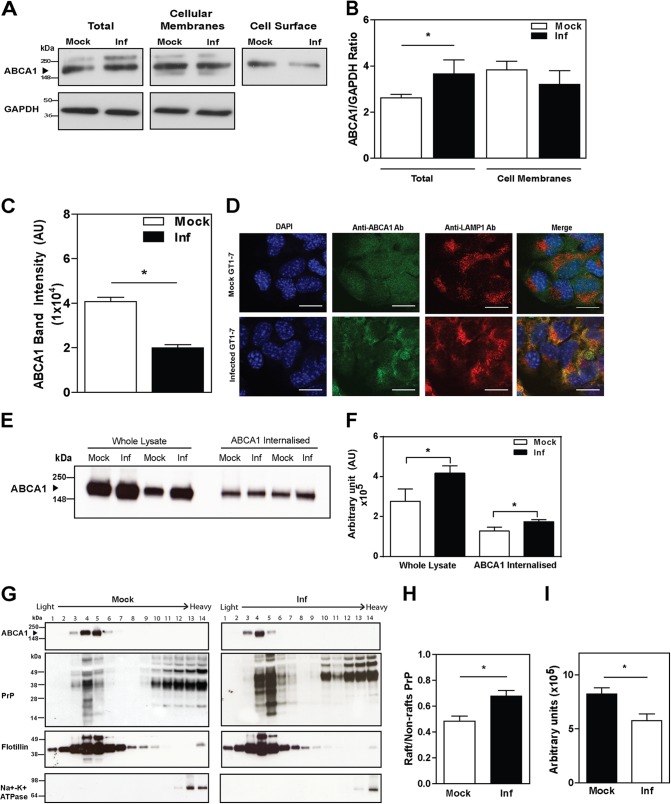
**Prion infection (*Inf*) impairs cholesterol efflux by redistributing ABCA1 from the cell surface and lipid rafts.** *A,* abundance of ABCA1 from whole lysate, cellular membrane fraction, and biotinylated ABCA1 at the cell surface in mock and prion-infected GT1-7 cells. *B,* densitometric quantification of total and membrane-associated ABCA1 protein in *A. C,* densitometric quantification of cell surface ABCA1 protein in *A. D,* intracellular localization of ABCA1 in mock (*top row*) and prion-infected GT1-7 cells (*bottom row*). *Scale bar,* 20 μm. *E,* ABCA1 internalization. Whole lysate and internalized ABCA1 were assessed using Western blotting. For internalized ABCA1, cell surface protein in mock- and prion-infected GT1-7 cells were labeled with sulfo-SS-biotin, followed by incubation at 37 °C for 30 min. Surface biotin was cleaved to detect the amount of ABCA1 internalized. Biotinylated protein was bound to streptavidin resin, and ABCA1 abundance was analyzed by Western blotting. *F,* densitometric quantification of whole lysate and internalized ABCA1 protein abundance in mock- and prion-infected GT1-7 cells. Mean ± S.D., *n* = 3, *, *p* < 0.05. *G,* distribution of ABCA1 and PrP in lipid rafts *versus* non-raft fractions of plasma membrane. *, *p* < 0.05. *H,* ratio of PrP abundance in lipid rafts *versus* non-raft fractions. *I,* ABCA1 abundance in lipid rafts of GT1-7 cells. *, *p* < 0.05.

Reduced cholesterol efflux on a background of elevated abundance of ABCA1 indicated that prion infection had affected ABCA1 functionality. A common reason for impaired ABCA1 functionality is mislocalization of ABCA1 away from the cell surface, resulting in a significant reduction of cholesterol efflux (31). To test this possibility, we evaluated the subcellular localization of ABCA1. Although prion infection increased the total amount of ABCA1 in the cells, the amount of ABCA1 associated with cellular membranes (both plasma membrane and intracellular membranes) was similar in mock- and prion-infected cells (Fig. 4, *A* and *B*). Moreover, the abundance of cell surface ABCA1 in prion-infected cells was reduced by 50% compared with mock-infected cells (Fig. 4, *A* and *C*) thus explaining the discrepancy between the ABCA1 level and the level of cholesterol efflux. More specifically, we demonstrated a significant increase of co-localization between ABCA1- and LAMP1-positive endosomes (Fig. 4*D*) in prion-infected cells. It appears that prion infection causes a redistribution of ABCA1 away from the cell surface into intracellular compartments.

To test this hypothesis, we measured the rate of ABCA1 internalization; the findings are shown in Fig. 4, *E* and *F*. In these experiments cell surface ABCA1 was first labeled with biotin; the reaction was stopped, and biotinylated ABCA1 was allowed to internalize. The biotin label was then removed from proteins remaining at the cell surface, leaving only the internalized proteins labeled with biotin. Although the amount of cell surface ABCA1 was reduced by prion infection (Fig. 4*A*), the amount of internalized ABCA1 was elevated (Fig. 4*E*, quantitation from several experiments is shown in Fig. 4*F*) indicating a higher rate of internalization.

Lipid rafts play an important role in both prion infection and ABCA1 functionality. On the one hand, lipid rafts are thought to be the conversion site where physiological PrP^C^ misfolds into PrP^Sc^. On the other hand, lipid rafts are the main source of cholesterol for ABCA1-dependent cholesterol efflux (32), and lipid rafts and ABCA1 exist in a dynamic equilibrium (33–35). We therefore investigated co-localization of PrP and ABCA1 in membrane fractions separated by Optiprep density gradient centrifugation; Flotillin-1 was used as a raft marker and Na^+^K^+^-ATPase as a non-raft marker. PrP was detected in both raft and non-raft fractions; consistent with previous reports (36), prion infection caused a considerable re-distribution of PrP from non-raft to raft fractions (Fig. 4, *G* and *H*). Distribution of ABCA1 in relation to rafts is likely to be cell-specific (37); in GT1-7 cells, we found that ABCA1 was localized in rafts in both mock- and prion-infected cells. In mock-infected cells, most ABCA1 was found in fractions 4–5, whereas in the infected cells, most ABCA1 was detected in lighter fractions 3–4 (Fig. 4*G*). Quantitative assessment shows that ABCA1 abundance in lipid rafts decreased in prion-infected cells (Fig. 4*I*). Thus, in GT1-7 cells, conversion of PrP^C^ into PrP^Sc^ led to the redistribution of PrP into rafts and of ABCA1 away from rafts. The decrease of ABCA1 in rafts occurred without elevation of ABCA1 abundance in non-raft fractions of the plasma membrane, supporting the hypothesis that prion infection causes internalization of ABCA1.

##### Reversal of the Effects of Prion Infection on ABCA1 and Cholesterol Efflux

**FIGURE 5.**
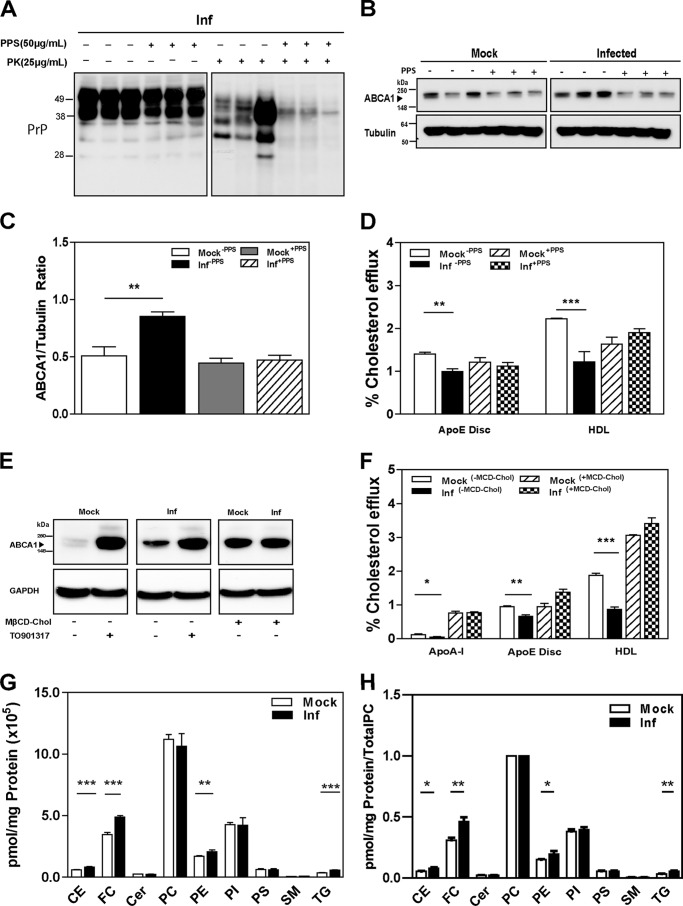
**Reversal of the effects of prion infection (*Inf*).** *A,* cells were treated with PPS (50 μg/ml) or vehicle continuously for 8 days. Western blots of cell lysate with and without PK treatment are shown after development with anti-PrP antibody. *B,* ABCA1 protein abundance was assessed by Western blotting comparing pre- and post-PPS treatment. *C,* densitometric quantification of ABCA1 protein abundance before and after treatment with PPS in mock- and prion-infected GT1-7 cells. **, *p* < 0.01. *D,* cholesterol efflux to apoE discs (15 μg/ml) and HDL (40 μg/ml) were measured in mock and infected GT1-7 cells, treated and untreated with PPS. **, *p* < 0.01; ***, *p* < 0.001. *E,* ABCA1 protein abundance after prion-infected and mock GT1-7 cells were loaded with cholesterol by incubating with 5 mm cholesterol-loaded methyl-cyclodextrin (*M*β*CD-Chol*). *F,* cholesterol efflux in mock- and prion-infected GT1-7 cells, incubated with or without cholesterol loaded methyl-cyclodextrin. *, *p* < 0.05; **, *p* < 0.01; ***, *p* < 0.001. *G* and *H,* lipidomic analysis for mock- and prion-infected GT1-7 cells; absolute values (*G*) or normalized to PC content (*H*). *CE,* cholesterol esters; *FC,* free cholesterol; *Cer,* ceramides; *PC,* phosphatidylcholine; *PI,* phosphatidylinositol; *PS,* phosphatidylserine; *SM,* sphingomyelin; *TG,* triglycerides.**, *p* < 0.01; ***, *p* < 0.001.

To directly connect changes in cholesterol efflux and ABCA1 abundance with prion infection, we performed “rescue” experiments. GT1-7 cells were treated with PPS, a compound that cures prion infection *in vitro* by disrupting the interactions between PrP and endogenous glycosaminoglycans at the cell surface, thereby reducing PrP^Sc^ accumulation (38). After treatment with PPS for 8 days at a noncytotoxic concentration, there was a significant reduction of PrP^Sc^ present in prion-infected cells (Fig. 5*A*). Furthermore, ABCA1 abundance in PPS-treated prion-infected cells was reduced to a level similar to mock-infected cells where there was no effect of PPS on ABCA1 abundance (Fig. 5, *B* and *C*). PPS also reversed the effect of prion infection on cholesterol efflux to apoE/POPC and HDL, and there was no effect of PPS on cholesterol efflux from mock-infected cells (Fig. 5*D*). Thus, increased ABCA1 abundance and impairment of cholesterol efflux were reversed when prion infection was cured.

Given that cellular cholesterol level affects both prion infection and abundance and functionality of ABCA1, we investigated how changes in cellular cholesterol would modulate the effects of prion infection on cholesterol efflux. Loading the cells with exogenous cholesterol using methyl-β-cyclodextrin·cholesterol complex resulted in an increase in ABCA1 abundance in both mock and infected cells eliminating the difference between prion-infected and mock-infected cells (Fig. 5*E*). Addition of exogenous cholesterol also increased cholesterol efflux to all acceptors in both infected and mock-infected cells eliminating the difference between the two (Fig. 5*F*). Thus, the effects of prion infection on ABCA1 abundance and cholesterol efflux are dependent on cell cholesterol content.

##### Effect of Prion Infection on Cellular Lipids

To investigate the physiological consequences of impairment of cholesterol efflux by prion infection, we performed lipidomic analysis on mock- and prion-infected GT1-7 cells. Fig. 5*G* presents data with different subclasses of lipids combined and categorized into seven main classes; full lipidomic analysis is presented in supplemental Table S1. In prion-infected cells, cholesterol esters, free cholesterol, phosphatidylethanolamine (PE), and triglycerides were significantly increased, although no changes were detected in other lipid classes when compared with mock-infected cells (Fig. 5*G*). When the abundance of different lipids was normalized to total PC level, again, in prion-infected cells cholesterol ester, free cholesterol, PE, and triglycerides were significantly increased, whereas other lipids remained unchanged (Fig. 5*H*).

##### Elevation of ABCA1 Reduces PrP^Sc^ Formation

**FIGURE 6.**
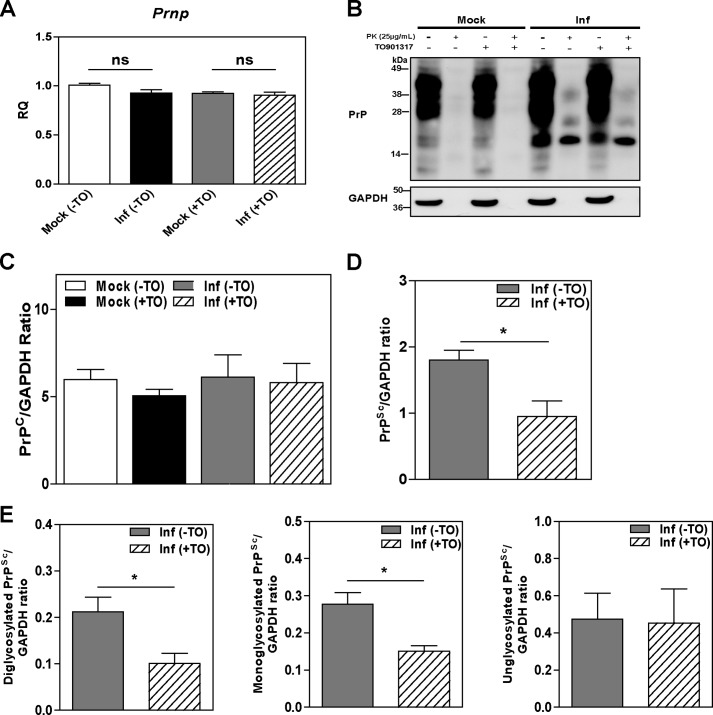
**LXR agonist TO901317 reduced PrP^Sc^ formation in GT1-7 cells.** *A,* effects of LXR agonist TO901317 on mRNA expression levels of *Prnp* as assessed by RT-PCR in mock- and prion-infected GT1-7 cells either treated (+) or untreated (−) with TO901317. *B,* PrP^C^ and PrP^Sc^ protein abundance was assessed by Western blot in TO901317 (*TO*)-treated (+) and -untreated (−) cells. *C–E,* densitometric quantification of PrP^C^ (*C*) and PrP^Sc^ (*D* and *E*) protein abundance in cells treated (+) or untreated (−) with TO901317. Total PrP^Sc^ abundance is shown in *D*, and the abundance of the individual glycosylated PrP isoforms is shown in *E*; ratios of specific PrP band to GAPDH are shown; for PK-treated samples GAPDH values in parallel samples untreated with PK were used. *, *p* < 0.05. *ns,* nonsignificant.

The effects of prion infection on cholesterol metabolism indicate the possibility that resisting these changes may affect prion infection. We tested whether levels of prion protein are affected by activation of LXR, a potent activator of ABCA1 expression and cholesterol efflux. When prion-infected cells were treated with the LXR agonist TO-901317, there were no observable changes in the level of prion gene (*Prnp*) mRNA (Fig. 6*A*) or in the level of PrP^C^ protein (Fig. 6, *B* and *C*). However, the total amount of PrP^Sc^ in infected cells was significantly reduced upon LXR agonist treatment (Fig. 6, *B* and *D*). The reduction of PrP^Sc^ affected heavier, presumably glycosylated, isoforms of PrP^Sc^ (Fig. 6*E*).

**FIGURE 7.**
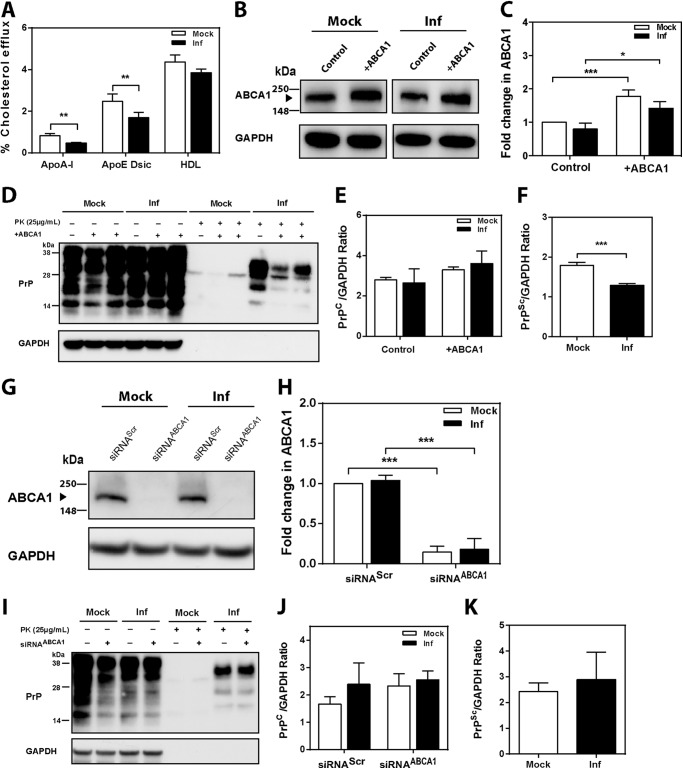
**Effect of ABCA1 overexpression and silencing on PrP^Sc^ formation in 3T3 cells.** *A,* cholesterol efflux to apoA-I (30 μg/ml), apoE discs (15 μg/ml), and HDL (40 μg/ml) from mock- or prion-infected 3T3 cells; cells were activated with 4 μm LXR agonist (mean ± S. D., *n* = 3). *Inf*, infected. **, *p* < 0.01 *versus* mock-infected cells. *B,* Western blot analysis of ABCA1 abundance in 3T3 cells after transient transfection with mock (*control*) and human ABCA1 plasmid (+*ABCA1*). *C,* relative change of ABCA1 abundance in mock- and prion-infected 3T3 cells after transfection with mock and ABCA1 plasmid. ABCA1 abundance was expressed as fold change compared with mock (*control*) (mean ± S.D., *n* = 3). *, *p* < 0.05; ***, *p* < 0.001. *D,* Western blot analysis of PrP^C^ and PrP^Sc^ in 3T3 cells transiently transfected with mock and human ABCA1 plasmids. *E* and *F,* densitometric quantification of PrP^C^ (*F*) and PrP^Sc^ (*G*) in 3T3 cells transfected with mock and ABCA1 plasmids. (mean ± S.D., *n* = 3). ***, *p* < 0.001 *versus* mock-infected 3T3 cells. *G,* Western blot analysis of ABCA1 abundance in 3T3 cells after transfection with scrambled (siRNA^Scr^) and mouse ABCA1-specific siRNA (siRNA^ABCA1^, final concentration 20 nm). *H,* relative protein fold change of ABCA1 in mock-infected and prion-infected 3T3 cells after transfection with siRNA^ABCA1^ or siRNA^Scr^. ABCA1 abundance was expressed as fold change compared with mock-infected cells (mean ± S.D., *n* = 3). ***, *p* < 0.001. *I,* Western blot analysis of PrP^C^ and PrP^Sc^ in mock- and prion-infected 3T3 cells transfected with siRNA^Scr^ (−) and siRNA^ABCA1^ (+). Densitometric quantification of PrP^C^ (*J*) and PrP^Sc^ (*K*) in mock- and prion-infected 3T3 cells transfected with siRNA^Scr^ and siRNA^ABCA1^.

Activation of LXR affects many genes; therefore, we corroborated findings of the experiments with LXR agonist by overexpressing and silencing ABCA1 by heterologous transfection. Because of the difficulty in transfecting GT1-7 cells with efficiency sufficient for testing cholesterol efflux, 3T3 mouse fibroblasts were infected with mouse prion and transfected either with ABCA1 or siRNA^ABCA1^. Infection of 3T3 cells with prions resulted in reduction of cholesterol efflux to apoA-I, apoE discs, and HDL similar to the effects seen in prion-infected neuronal cells (Fig. 7*A*). In contrast to neuronal cells, we did not observe an elevation of cellular ABCA1 levels as a result of prion infection (Fig. 7, *B* and *C*). Overexpression of heterologous ABCA1 increased the abundance of ABCA1 in both infected and mock-infected cells (Fig. 7, *B* and *C*). Overexpression of ABCA1 did not affect levels of PrP^C^, but a statistically significant reduction in the level of PrP^Sc^ was observed (Fig. 7*D* and quantitation in *E* and *F*), similar to the effects of an LXR agonist.

Transfection of cells with siRNA^ABCA1^ resulted in an almost complete elimination of ABCA1 from both mock-infected and prion-infected cells (Fig. 7, *G* and *H*). Silencing of ABCA1 did not affect the levels of PrP^C^ or PrP^Sc^ (Fig. 7*I* and quantitation in *J* and *K*).

## DISCUSSION

It is established that an imbalance of neuronal cholesterol homeostasis plays a key role in pathogenesis of many neurodegenerative diseases, including prion disease. However, the mechanisms by which prion infection affects cholesterol homeostasis in neuronal cells remains unclear. The main finding of this study is that prion infection impairs cholesterol efflux from neuronal cells despite elevated levels of ABCA1 and unchanged levels of ABCG1. Mechanistically, the effects on cholesterol efflux were explained by displacement of ABCA1 from lipid rafts and the cell surface to intracellular compartments. These effects were reversed when prion infection was cured or when the cells were loaded with cholesterol. Physiologically, prion infection led to the accumulation of free and esterified cholesterol, PE, and triglycerides in neuronal cells. Furthermore, overexpression of ABCA1 reduced the severity of prion infection. The identification of PE accumulation is significant given the recent findings that this lipid alone can act as a sole cofactor in forming infectious prion molecules from highly purified recombinant sources of PrP (39, 40).

**FIGURE 8.**
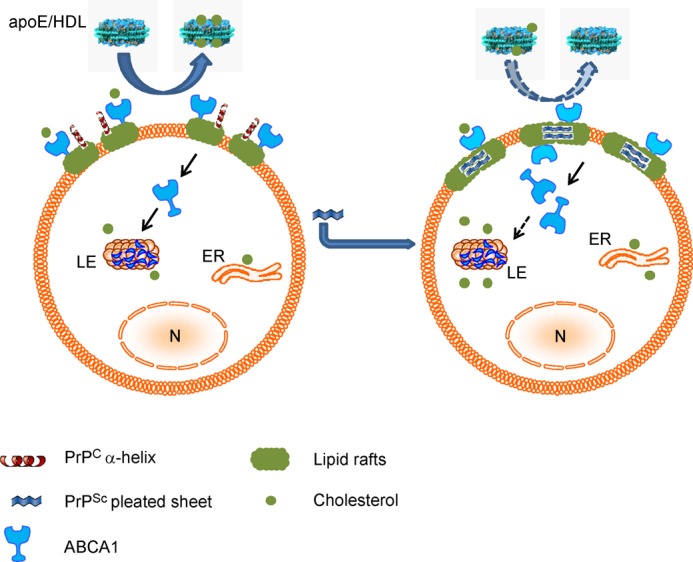
**Proposed mechanism of prion affecting cholesterol efflux.** In uninfected neuronal cells (*left*), both ABCA1 and PrP^C^ proteins are localized to rafts. Monomeric PrP^C^ does not interfere with the capacity of ABCA1 to remove cellular cholesterol to the main cholesterol acceptor in the cerebrospinal fluid, apoE-based HDL. The formation of PrP^Sc^ in the rafts as a result of prion infection may change the structure of lipid rafts, transforming them into a larger, more rigid structure (*right*). This can lead to the displacement of ABCA1 from rafts and the cell surface, ultimately reducing ABCA1 functionality and/or preventing removal of cholesterol from rafts. Reduction of cholesterol efflux leads to accumulation of cholesterol in the cells, which partitions into rafts stabilizing the platform for accumulation and further conversion of PrP^C^ to PrP^Sc^. Thus, conversion of prion protein and reduction of cholesterol efflux may form a vicious circle contributing to the development of the prion disease. *LE,* late endosomes; *ER,* endoplasmic reticulum; *N*, nucleus.

It has been thought that prion infection requires high levels of cellular cholesterol because of its reliance on lipid rafts. PrP^C^ is partly located within lipid rafts, and this is where conversion to PrP^Sc^ is thought to take place. It is also known that these microdomains are very sensitive to cell cholesterol content. It was speculated that localization of PrP^C^ in rafts is necessary for conversion because it allows for a high local concentration of PrP^C^ and for a “detergent-like” environment, both factors favoring polymerization and conversion of prion proteins (41). ABCA1 not only functions to reduce cellular cholesterol content, but its overexpression can also specifically reduce the number of rafts, whereas increased abundance of rafts reduces the abundance of ABCA1 at the plasma membrane (33, 34, 42). Based on these considerations, we propose the following mechanistic model of how and why prion infection affects cholesterol efflux (Fig. 8). In uninfected neuronal cells, both ABCA1 and PrP^C^ proteins are localized in rafts. Monomeric PrP^C^ does not interfere with the capacity of ABCA1 to remove cellular cholesterol to the main cholesterol acceptor in the cerebrospinal fluid, apoE-based HDL. The formation of PrP^Sc^ in the rafts as a result of prion infection may change the structure of lipid rafts, transforming them into larger, more rigid structures. This can lead to the displacement of ABCA1 from rafts and the cell surface to the intracellular compartments, presumably late endosomes, ultimately reducing ABCA1 functionality and resisting removal of cholesterol from rafts. Reduction of cholesterol efflux leads to accumulation of cholesterol in the cells, which partitions into rafts stabilizing the platform for the accumulation and further conversion of PrP^C^ to PrP^Sc^. Thus, conversion of the prion protein and reduction of cholesterol efflux may form a vicious cycle contributing to the progression of the prion disease. Regulation of the delivery arm of cholesterol homeostasis is affected by prions (12), limiting the compensatory response of the host cells to attempts to stimulate cholesterol efflux by up-regulating the key element of the removal arm of cholesterol homeostasis, ABCA1. Post-translational mechanisms are apparently responsible for the elevation of the ABCA1 levels in neuronal cells at prion infection, likely reflecting an accumulation of cholesterol in these cells. Elevation of ABCA1 was not observed in prion-infected fibroblasts, possibly because these cells usually do not accumulate excessive cholesterol. Regulation of ABCA1 functionality by its mislocalization is a well established phenomenon exploited by pathogens, such as HIV (42). In prion disease, when rafts are “occupied” with PrP^Sc^, ABCA1 is dysfunctional. However, loading cells with free cholesterol overcomes this prion-inflicted restriction, likely by providing sufficient cholesterol and a cholesterol-rich environment required for ABCA1 to be stable and functional (37), thus elevating cholesterol efflux to the level seen in uninfected cells. Interestingly, prion disease specifically targets ABCA1, but affects cholesterol efflux not only to the lipid-free apoA-I, which is the preferred substrate of ABCA1, but also to HDL and lipidated apoE, which are preferred substrates for two other transporters, ABCG1 and SR-BI. However, this seems to be a peculiarity of the neuronal cells as it was demonstrated before (28) and confirmed in this study that SR-BI is not expressed in neuronal cells. It was also demonstrated that in neuronal cells ABCA1 along with ABCG1 supports cholesterol efflux to the lipidated acceptors, including apoE-based HDL (29). Given the established role of ABCA1 in pathogenesis of other neurodegenerative disorders such as Alzheimer disease, ABCA1 seems to be the most important transporter regulating cellular cholesterol homeostasis in CNS.

There are several implications of this study for the diagnosis and treatment of prion disease. First, if deficiency of cholesterol efflux plays a role in pathogenesis of prion infection, it may also be a risk factor. It is tempting to speculate that subjects with impaired cholesterol efflux may be more susceptible to prion infection or for spontaneous conversion of PrP^C^ to PrP^Sc^. Second, treatment with an LXR agonist and overexpression of heterologous ABCA1, treatments that stimulate both cholesterol efflux and ABCA1 abundance, reduce PrP^C^ to PrP^Sc^ conversion, thus warranting further consideration as an option for treatment of prion disease.

In conclusion, we have demonstrated that prion infection post-translationally modifies the cholesterol efflux pathway by displacing ABCA1 from lipid rafts. This mechanism is not dissimilar to that used by HIV, which also requires cholesterol for its replication (42). Prions and HIV are very different pathogens; however, their requirement for cholesterol may be a common element that is required for successful propagation of infection. Remarkably, they target the same pathway and use similar mechanisms to secure cholesterol required for their replication.

## Footnotes

7 The abbreviations used are:

PrP^C^: cellular prion protein
ABCA1: ATP-binding cassette transporter type A1
ABCG1: ATP-binding cassette transporter type G1
apoA-I: apolipoprotein A-I
apoE: apolipoprotein E
LXR: liver X receptor
POPC: 1-palmitoyl-2-oleylphosphatidylcholine
PE: phosphatidylethanolamine
PPS: sodium pentosan polysulfate
PrP: prion protein
SR-BI: scavenger receptor class B, type I.
